# Induction of Systemic Resistance against Insect Herbivores in Plants by Beneficial Soil Microbes

**DOI:** 10.3389/fpls.2017.01816

**Published:** 2017-10-20

**Authors:** Md. Harun-Or Rashid, Young R. Chung

**Affiliations:** Division of Applied Life Science (BK21 Plus), Plant Molecular Biology and Biotechnology Research Center, Gyeongsang National University, Jinju, South Korea

**Keywords:** beneficial soil microbes, induced systemic resistance, insect herbivores, priming, signaling pathway

## Abstract

Soil microorganisms with growth-promoting activities in plants, including rhizobacteria and rhizofungi, can improve plant health in a variety of different ways. These beneficial microbes may confer broad-spectrum resistance to insect herbivores. Here, we provide evidence that beneficial microbes modulate plant defenses against insect herbivores. Beneficial soil microorganisms can regulate hormone signaling including the jasmonic acid, ethylene and salicylic acid pathways, thereby leading to gene expression, biosynthesis of secondary metabolites, plant defensive proteins and different enzymes and volatile compounds, that may induce defenses against leaf-chewing as well as phloem-feeding insects. In this review, we discuss how beneficial microbes trigger induced systemic resistance against insects by promoting plant growth and highlight changes in plant molecular mechanisms and biochemical profiles.

## Introduction

Plants are primary producers of organic nutrients, which nurture all heterotrophic organisms in the natural ecosystem. Especially in soils, plants play crucial roles in a complex food web system where many microorganisms utilize the plant’s supplied nutrients in exchange for a limiting resource (Paul, 2007). In the interactions between soil microorganisms and host plants, the root system is the predominant host, which deposits up to 40% of photosynthetic carbon into the root zone. The rhizosphere is the greatest energy-rich zone in the ecosystem (Bais et al., 2006). The beneficial rhizosphere microbiota includes PGPR and PGPF. These genera enhance plant growth and improve health in many different ways (Lugtenberg and Kamilova, 2009; Shoresh et al., 2010). A wide range of beneficial microbes also provide plants with important capabilities, such as enriched nutrient uptake, growth promotion, and defense from pathogens and insects (Lugtenberg and Kamilova, 2009; van de Mortel et al., 2012; Lareen et al., 2016).

Beneficial microorganisms in plant roots can improve plant health by priming the entire plant to increase the defense against various pathogens and insect herbivores by the mechanism of ISR (Pieterse et al., 2014). ISR is activated by non-pathogenic bacteria in SA-independent and -dependent manners, and somewhat intersects with the JA/ET pathway. SAR is stimulated by necrotizing pathogens and a SA-dependent signaling pathway, and results in enhanced level of SA and activation of PR proteins (Conrath et al., 2002; Hammerschmidt, 2009; Van der Ent et al., 2009). *Pseudomonas fluorescens* SS101 induces resistance against some plant pathogens such as *P. syringae* pv. *tomato* and the herbivorous insect pest *Spodoptera exigua* (van de Mortel et al., 2012). Hormone pathways and molecules participating in the recruitment of particular groups of microorganisms following foliar herbivore attack and defense stimulation have been reported (de Roman et al., 2011; Doornbos et al., 2011; Yang et al., 2011; Yi et al., 2011; Lakshmanan et al., 2012).

Jasmonic acid and SA are plant hormones that are central in coordinating the complex signaling pathways. Other hormones, such as auxin, ET, CK, ABA and GA, can also modulate signaling pathways during interactions between plants and biotic factors, pathogens and insects (Robert-Seilaniantz et al., 2011; Meldau et al., 2012; Pieterse et al., 2012; Giron et al., 2013). Therefore, plant–microbe and plant–insect interactions are connected through molecular pathways. Induction of hormone signaling pathways depends on insect feeding behaviors (Pineda et al., 2010). The phytohormones ET, JA, and SA can regulate symbiosis and mediate ISR triggered by beneficial microbes in the interactions occurring between non-pathogenic rhizosphere microbes and plants (De Vleesschauwer and Höfte, 2009; Zamioudis and Pieterse, 2012). Likewise, several rhizobacteria induce biochemical changes that trigger ISR in plants against insect herbivores (van de Mortel et al., 2012; Wielkopolan and Obrepalska-Steplowska, 2016; Zebelo et al., 2016). However, little is known of the tri-trophic level interaction between plants, insects, and microbes (Pineda et al., 2010).

The present review focuses on the molecular mechanisms and biochemical profiles involved in the ISR elicited by beneficial microbes against insect herbivores and highlights recent findings that will help stimulate research on the tri-trophic level interaction.

## Effect of Plant Health Improvement by Soil Microbes on Interaction with Insects

**FIGURE 1:**
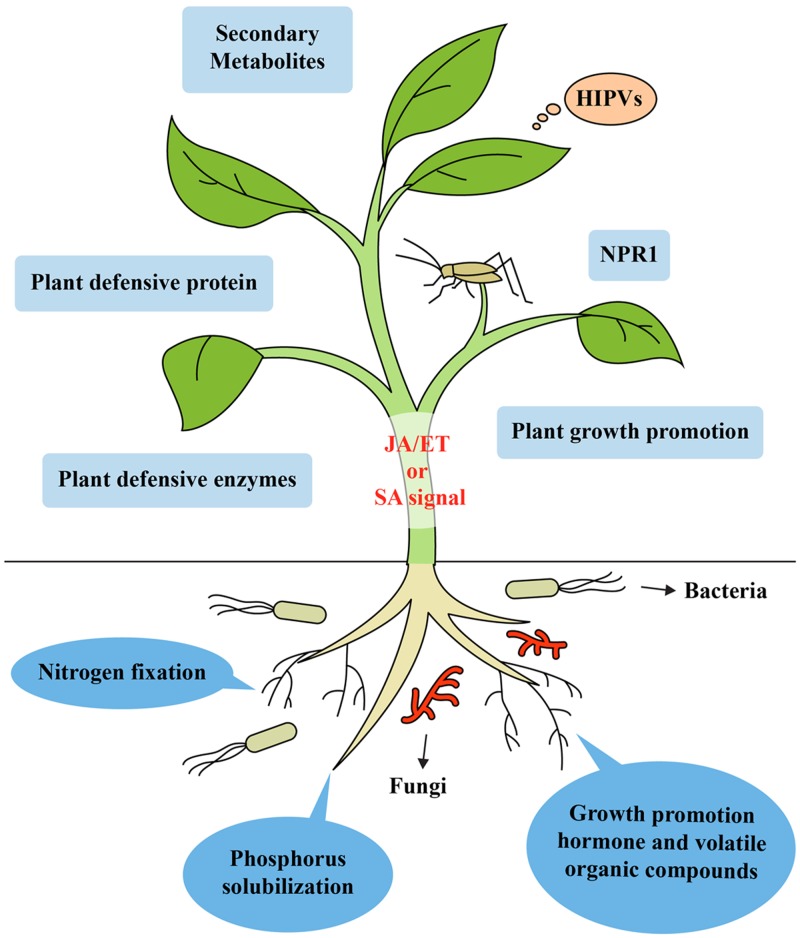
Model of induced systemic resistance in plants against insect herbivores by beneficial soil microbes like rhizobacteria and fungi. Recognition of unique MAMPs of microbes by plant receptors leads to the generation of a distinct ISR signal in the roots. Mobilization from the roots to shoots triggers ISR in the leaves by simultaneously activating the SA-, JA-, and ET-dependent signaling pathways. These signaling pathways lead to the expression of genes encoding NPR1, secondary metabolites, enzymes, plant defensive protein, and VOCs. Microbes can improve plant health by increasing the uptake and concentration of a variety of nutrients like phosphorus, solubilizing plant nutrients unavailable to plants in certain soils (e.g., rock phosphate) and fixing atmospheric nitrogen, producing secondary metabolites, enzymes, volatile organic compounds, herbivore induced plant volatiles and growth promoting hormone, which can trigger ISR in plants against insect herbivores.

Beneficial soil microorganisms, such as PGPR and PGPF, can improve plant health by fixing atmospheric nitrogen, solubilizing plant foods otherwise unobtainable in special types of soils like rock phosphate and increasing the uptake of nutrients (Spaink, 2000; Harrison, 2005). Several microbes have the capacity to biosynthesize plant hormones including IAA, cytokinins, auxins and gibberellins, which are essential for promoting growth (Van Loon, 2007; Contreras-Cornejo et al., 2009). Some rhizobacteria can enhance plant growth via the biosynthesis of secondary metabolites, volatile compounds and enzymes and also increase plant photosynthesis by modulating endogenous sugar and ABA signaling (Zhang et al., 2008; Vacheron et al., 2013). Along with their plant growth-promoting properties, rhizobacteria can increase plant health and trigger resistance to plant pathogens and insect herbivores by inducing systemic defense responses (Van Wees et al., 2008; Segarra et al., 2009; Hossain et al., 2016). These effects of soil microbes on improved plant growth affect plant–insect interactions, resulting in an enhanced food supply for insects. Furthermore, improved nutrient composition can increase nutritional value of plants, which affects insect performance at certain trophic levels (Schoonhoven et al., 2005; Bukovinszky et al., 2008). Different insects can benefit from the greater availability of nutrients in plant cells (Schoonhoven et al., 2005). Beneficial microbes enable the re-growth of tissues after herbivory due to increased nutrient and water uptake, which stimulates plant tolerance. This is reflected in detriments to plant yield or plant biomass in the presence of insects (Kula et al., 2005; Herman et al., 2008; Kempel et al., 2009). Moreover, greater photosynthesis efficiency enables beneficial microbes to convert more light energy, which allows the generation of an ISR against phloem feeder insects, which can compensate for the loss of plant energy (Valenzuela-Soto et al., 2010). Thus, microbes can improve plant health in various ways that include the increased uptake of nutrients, and the production of secondary metabolites, enzymes, volatile organic compounds, and growth hormones. All these directly or indirectly trigger ISR in plants against insect herbivores (**Figure 1**). However, these significant features have not been considered accurate enough to elucidate mechanisms of plant-microbe- insect interactions.

## Role of Phytohormones in ISR Against Insect Herbivores Mediated by Beneficial Microbes

Salicylic acid, ET, and JA are key plant hormones that regulate ISR during tri-trophic interactions (Shavit et al., 2013). These hormone-dependent pathways can regulate defense responses in different ways against specific types of attacking insects (Van Oosten et al., 2008). JA-mediated defenses are activated against herbivorous insects (Kessler et al., 2004; De Vos et al., 2005; Zheng et al., 2007; Howe and Jander, 2008; Van Oosten et al., 2008). JA signaling is the main ISR pathway activated to defend plants against leaf chewing insect pests, and is triggered by root-associated microorganisms (Van Oosten et al., 2008; Pineda et al., 2010; Jung et al., 2012). *Arabidopsis* roots treated with rhizobacteria induce resistance to chewing insects through the increased expression of JA-dependent gene *LOX2* and the JA- and ET-dependent genes, *PDF1.2* and *HEL* (Pangesti et al., 2015a). The colonization of plant roots by rhizobacterium *P. simiae* WCS417r elicits higher expression of the JA/ET-dependent ORA59-branch than the JA-dependent MYC2 branch, and triggers ISR against leaf-chewing insects (Pangesti et al., 2016). Root colonization of cotton plants by PGPR induces higher levels of JA, an octadecanoid-derived, defense-related phytohormone and JA-related genes, which may confer resistance against the leaf-chewing insect, *S. exigua* (Zebelo et al., 2016).

Using different mechanisms, *Bacillus subtilis* PGPR induces resistance against the phloem insect whitefly on tomato plants (*Solanum lycopersicum*), increased expression of both JA-independent genes (including photosynthetic genes, phenyl-propanoid and terpenoid biosynthetic pathways genes) and JA-dependent genes including proteases and proteinase inhibitor coding genes (Valenzuela-Soto et al., 2010). Pineda et al. (2012) reported that *Arabidopsis* roots colonized by *P. fluorescens* WCS417r have enhanced susceptibility to the phloem-feeding aphid *Myzus persicae*, although treated plants showed stronger expression of *LOX2* and *PDF1.2* gene following insect attack. These studies show that different rhizobacteria genera including *Bacillus* and *Pseudomonas* have different effects against phloem-feeding insects. Further studies are needed.

*Pseudomonas fluorescens* SS101 activates ISR through SA-dependent mechanisms, although most rhizobacteria facilitate ISR through JA- and ET-dependent mechanisms (van de Mortel et al., 2012). Niu et al. (2011) also showed that PGPR-triggered ISR is dependent on both the JA/ET- and SA- signaling pathways. It is assumed that the MAMPs of different beneficial microbes might be recognized by plant receptors leading to specific hormonal signals produced in the roots. MAMPs of beneficial microbes including flagellin, secondary metabolites and lipopolysaccharides activate MAMP-triggered immunity (MTI) and modulate hormonal signals in plants (Jacobs et al., 2011; Hermosa et al., 2012; Zamioudis and Pieterse, 2012). For example, *B. amyloliquefaciens* S499 produces lipopeptides, which lead to enhanced expression of defense-related genes *lipoxygenase D* and *F* (*LOXD, LOXF*) that induced ISR in tomato plants (Cawoy et al., 2014). Another lipopeptide producing endophyte, *B. amyloliquefaciens* strain Blu-v2, elicits ISR in plants against fall armyworms (Li et al., 2015). The expression of ISR against pathogens and insects requires responsiveness to the SA- and JA/ET-signaling pathways and is dependent on ‘non-expressor of pathogenesis-related genes1’ (Pieterse et al., 1998; Mewis et al., 2005; Segarra et al., 2009; van de Mortel et al., 2012). Mobilization of distinct signal from the roots to the shoots triggers ISR in the leaves by simultaneously activating SA-, JA-, and ET-dependent signaling pathways. These signaling pathways may lead to the expression of genes encoding NPR1, which can trigger ISR against insect herbivores (**Figure 1**). Unfortunately, how the MAMPs of beneficial microbes modify phytohormone signaling pathways in the plants during interaction with insects is not completely understood. Further studies are required to clarify how microbial MAMPs affect plant hormonal signals by infestation of insect herbivores.

## Induced Production of Defense-Related Compounds in Plant-Microbe-Insect Interactions

Root colonization by beneficial microbes can induce biosynthesis of plant defense-related compounds against insects through various plant hormonal signaling pathways (van de Mortel et al., 2012; Pangesti et al., 2016). The production of defense-related chemical compounds, such as flavonoids, lignin and other secondary metabolites, that produce effective defense against a wide range of plant pathogens and insect herbivores is regulated in the JA/ET and SA pathways (Pauwels et al., 2009; Valenzuela-Soto et al., 2010; Campos et al., 2014; Mejía et al., 2014). Among these compounds, camalexin and glucosinolates have important roles in plant defenses against pathogenic microbes, leaf chewers and sap sucking insect herbivores (Mewis et al., 2006; Kim et al., 2008; Kusnierczyk et al., 2008; Clay et al., 2009; Müller et al., 2010). The biosynthesis of camalexin and glucosinolates, which triggers ISR against leaf-chewing insects, is increased by rhizobacterial colonization of the roots via the JA and ET pathways (Pangesti et al., 2016). The colonization of *Arabidopsis* roots by *P. fluorescens* SS1011 can also induce the biosynthesis of these two compounds that enhance ISR against the lepidopteron insect pest, *S*. *exigua*, through an SA signaling pathway (van de Mortel et al., 2012). Thus, colonization of roots by different rhizobacterial species could induce the production of the same defense compounds, such as camalexin and glucosinolates, via different signaling pathways.

Flavonoids are well-known plant secondary metabolites that frequently act as insect feeding inhibitors and pigments, to promote pollination by attracting insects (Schoonhoven et al., 2005). These compounds are also found in root exudates and are essential in root colonization of rhizobacteria (Ferguson and Mathesius, 2003; Steinkellner et al., 2007; Dennis et al., 2010; Zamioudis and Pieterse, 2012). Herbivory of the cabbage butterfly (*Pieris rapae*) and caterpillar (*Helicoverpa armigera*) can activate a group of JA-signaling pathways controlled by the transcription factor MYC2 that positively regulate the biosynthesis of flavonoids and anthocyanin, which produces resistance to the insect pests (De Vos et al., 2005; Dombrecht et al., 2007; Verhage et al., 2011). Fertilization of a microorganism-based product containing *Bacillus* and actinomycetes increases the expression of transcription factors such as *TT8, EGL3, MYB12, MYB114* and *MYB113*, which activate the genes for flavonoid biosynthesis resulting in the accumulation of flavonoids in *Arabidopsis* plants (Ali and McNear, 2014). The flavonoid tricin that is extracted from bluegrass infected with the endophytic fungus, *Neotyphodium typhnium*, has insecticidal activity against mosquito larvae (Ju et al., 1998). Tricin also reportedly inhibits infestation of brown planthopper in a resistant rice cultivar (Bing et al., 2007). Recent studies have implicated tricin in biosynthesis of lignin in monocots (Lan et al., 2015; Li et al., 2016).

Among the other secondary metabolites, the most common group of defensive compounds are phenolic compounds. They are important in the resistance strategy against pathogenic microbes and herbivorous insects (Sharma et al., 2009; Rani and Jyothsna, 2010; Mazid et al., 2011; War et al., 2011). Phenolic compounds are accumulated by PGPR. They are directly toxic and/or produce a hypersensitive response (HR) in plants (Singh et al., 2015; Kiprovski et al., 2016). Colonization of rice roots by *P. fluorescens* WCS374r induces ISR, which enhances accumulation of phenolic compounds (De Vleesschauwer et al., 2008). Lignin is an important phenolic. It is a complex phenolic heteropolymer that confers resistance against attack by herbivorous insects (Barakat et al., 2010). Increased lignin content in the plant cell wall can physically limit the entrance and feeding of insect herbivores by increasing leaf hardiness (Johnson et al., 2009). The biosynthesis of lignin and different oxidative phenols that participate in plant defense against insect pests is catalyzed by PPO and POD (Bhonwong et al., 2009; Gulsen et al., 2010). *P. fluorescens* strains Pf1, TDK1 and PY15 display ISR against the leaffolder larvae (*Cnaphalocrocis medinalis*) by the activation of PPO in rice plants (Saravanakumar et al., 2008). Treatment of *Arabidopsis* with beneficial microorganisms induces the expression of lignin pathway genes and results in the increased lignin content in leaves (Ali and McNear, 2014). Endophytic colonization by a foliar fungus increases the lignin content of leaves, which reduces the damage caused by pathogens and herbivore attack (Mejía et al., 2014). The mechanism of induced resistance against insects by beneficial soil microbes related with lignin biosynthesis remains unclear.

Gossypol is a phenolic sesquiterpenoid aldehyde that confers resistance to infestation by many chewing and sucking insect pests belonging to Aphididae, Miridae, Tetranychidae, Thripidae, and caterpillars. Especially, the host infestation capability of *Heliothis* and *Helicoverpa* (Noctuidae) larvae is suppressed by antibiosis or by aversion to cotton because of the high amount of gossypol in these plants (Syed et al., 2003; Du et al., 2004; Stipanovic et al., 2006). Exogenous application of JA to cotton plants can increase the level of gossypol, which reduces the growth and development of the mealybug, *Phenacoccus solenopsis* (Zhang et al., 2011). Treatment with *Bacillus* spp. can induce the expression of JA-related genes *GhLOX1*, *GhAOS* and *GhOPR3*, which initiates transcription of gossypol biosynthesis genes including the (+)-δ- cadinene synthase (CAD1) gene family (*Cdn1- A*, *CAD1-C1*, *Cdn1-C3*, and *Cdn1-C14*) to reduce herbivory by *S. exigua* larvae. The induced resistance of cotton plants against *S. exigua* might be due to the enhanced level of gossypol (Wu et al., 2010; Zebelo et al., 2016). Unlike other secondary metabolites, proteins like lipoxygenase (LOX) and jacalin-related lectin are associated with numerous defense related processes, which include formation of cell wall structure, stress adaptation and resistance to pathogens and insects in several crops (Pauwels et al., 2009; Tong et al., 2012). LOX has a prominent and direct role in stimulating plant defense by producing protease inhibitors and oxidative enzymes (Mao et al., 2007). *P. fluorescens* triggers ISR against the leaffolder larvae in rice plants by the activation of several enzymes including LOX, chitinases and trypsin inhibitors (Commare et al., 2002; Saravanakumar et al., 2007, 2008). The jacalin-related lectin Orysata reportedly displays insecticidal activity that protects plants against different types of insects (Al Atalah et al., 2014). The lectin is induced in soybean plants during interaction with bacterial pathogens including *Xanthomonas axonopodis* pv. *glycines*, *P. syringae* pv. *Tomato*, and *B. amyloliquefaciens* KPS46 (Buensanteai et al., 2009). However, the mechanism of induced production of this plant defensive protein following insect infestation by beneficial microbes is unknown. On the basis of recent advances in defense-related chemicals with ISR against insect herbivores, we suggest that plant defenses against insect herbivores can be induced by beneficial soil microbes through biochemical and physiological changes in plant cells. For example, the induced production of a chemical, such as the flavonoid tricin, is not only a chemical inhibitor of insects. Rather, it may be linked to physical modifications of cell wall by lignification. Beneficial soil microbes may induce reactions that lead to the production of both chemical and physical barriers to the infestation of plants by insect herbivores. We are only at the early stage of understanding how beneficial soil microbes modulate and regulate plant defenses against insect herbivores through metabolic changes. Further knowledge will require studies of the molecular mechanisms in tri-trophic levels. This understanding will inform the development of strategies for efficient biological pest management.

## Bacterial Volatile Organic Compounds and Herbivore-Induced Plant Volatiles in Plant Defense

Ryu et al. (2004) first showed that VOCs including 2, 3-butanediol and acetoin produced by PGPR *Bacillus* species initiate ISR that is dependent on ET and independent of the JA or SA signaling pathways in *Arabidopsis*. VOCs are also produced upon infestation of plants by insects. These are termed HIPVs. HIPVs can protect plants directly by deterring, repelling or poisoning the herbivores, and may act indirectly by enticing natural enemies of the attackers (Maffei, 2010; Aartsma et al., 2017; Martorana et al., 2017). Production of HIPVs is facilitated primarily by an interplay of the JA, SA, and ET pathways (Koornneef and Pieterse, 2008; Van der Ent et al., 2009; Jung et al., 2012). Infestation of several leaf chewing insects initiates the expression of terpene genes that are dependent on JA signaling, and which might play a role in insect–plant interactions (Dombrecht et al., 2007; Dicke and Baldwin, 2010; Verhage et al., 2011; Hong et al., 2012). Rhizobacterial treatment might enhance the biosynthesis of HIPVs. Colonization of *Arabidopsis* roots by *P. fluorescens* WCS417r increases the transcription of JA-dependent genes to produce plant volatiles upon caterpillar attack (Pangesti et al., 2015a). In addition, treatment with this bacterium can repress the release of aromatics including methyl salicylate, lilial, and terpene (*E*)-α- bergamotene by decreasing the expression of the terpene synthase genes *TPS03* and *TPS04* upon caterpillar attack. This results in the attraction of more parasitoids of the caterpillar to the caterpillar-attacked plants, which produces an indirect plant defense against the attacking herbivores (Pangesti et al., 2015b). This highlights the important role of VOCs in both direct and indirect plant resistance strategies against insect herbivores (Schausberger et al., 2012; Kamolsukyunyong et al., 2013). The collective knowledge supports the view that enhanced production of VOCs and HIPVs in association with beneficial soil microbes should be further developed to yield innovative tactics to control insect herbivores in an effective and environmentally friendly way.

## Hypersensitive Response in ISR Against Insect Herbivores

Microbe-mediated ISR that occurs upon insect infestation and pathogen infection includes HR-type reactions, elevated cell wall or apoplastic peroxidase activity, callose deposition and hydrogen peroxide (H_2_O_2_) accumulation (Conrath, 2006; Valenzuela-Soto et al., 2010; Niu et al., 2011; Rahman et al., 2015). Insect feeding induces oxidative stress responses that are essential elements of plant defense against the attacking insects. Likely, the biosynthesis of reactive oxygen species (ROS) and consequent cell death leads to systemic resistance in pathogen-infected plants (Jones and Dangl, 2006; Mur et al., 2007). ROS detoxification might reduce antioxidant levels, but increases poisonous oxidation elements in soybeans infested with corn earworm (Bi and Felton, 1995). ROS and local cell death are important measures employed by plants to protect themselves against the phloem sap feeding green peach aphid (Lei et al., 2014). Increased levels of H_2_O_2_ and other ROS in plants can directly kill insects by causing intestinal destruction. The mortality of green peach aphid following consumption of artificial diets containing H_2_O_2_ also supports the hypothesized effects of ROS (Liu et al., 2010). Accumulation of H_2_O_2_ enhances the protection against the phloem sap sucking brown planthopper (*Nilaparvata lugens*) in rice (Zhou et al., 2009). Programmed cell death (PCD) is a major plant defense factor against insect herbivores including aphids. PCD manipulates the nutritional quality of the host in plant–microbe interactions (Goggin, 2007; Mur et al., 2007). Rhizobacterial stimulation of LOX activates the oxylipin pathway to change fatty acids into reactive hydroperoxides, which can be further modified into diverse defense metabolites (Shah, 2005; Cawoy et al., 2014). Exposure of *Arabidopsis* roots with the endophytic bacterium, *B. velezensis* YC7010, can induce systemic resistance to aphids due to the increased accumulation of H_2_O_2_, cell death and deposition of callose in leaves (Rashid et al., 2017). The collective data indicate that ROS accumulation in plants interacting with microbes is an early defense response against insect predation. However, higher accumulation of ROS in plants may have detrimental effects (Walz et al., 2002). Du et al. (2015) showed that ROS scavengers, such as peroxidases, can obviate the detrimental effects and can induce defense against the brown planthopper in resistant rice cultivars. It is conceivable that alterations in redox status resulting from higher levels of ROS scavengers in plants courtesy of beneficial microbes might contribute to ISR upon insect infestation. Additional HR studies in terms of redox status to elucidate the mechanism of plant defense in the interaction between microbes, plants, and insects.

## Modulation of Host Immunity and Primed Enhanced ISR Against Insect Herbivores by Beneficial Soil Microbes

**FIGURE 2:**
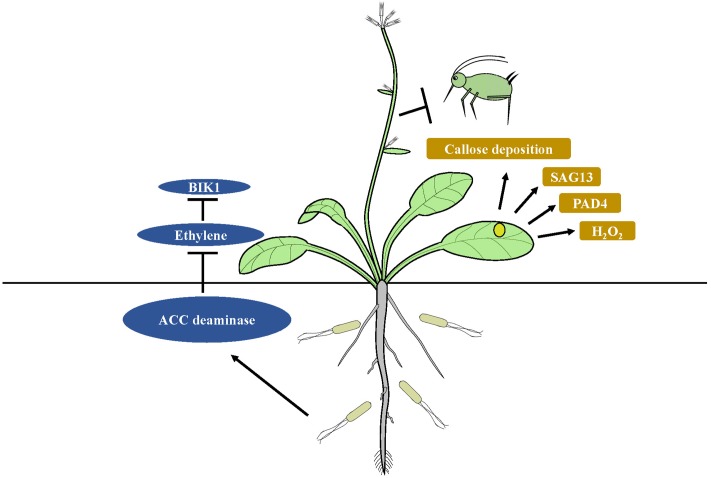
Model of suppression of host immunity by ISR-inducing microbes. Degradation of the ethylene precursor ACC by bacterial ACC deaminase, which suppresses ET-mediated immune responses like *BIK1*, results in higher expression levels of *PAD4* and *SAG13* in microbe treated plants. Enhanced expression of *PAD4* triggers more rapid H_2_O_2_ accumulation, cell death and callose deposition in plants, which can trigger ISR in the plants against insect herbivores.

The immune system of plants features specified pattern-recognition receptors (PRRs) that identify common microbial compounds, such as fungal chitin or bacterial flagellin. The patterns are termed MAMPs and PAMPs. Recognition of PAMPs or MAMPs by receptors is the first step in the basal plant defense response, which is collectively termed MTI (Jones and Dangl, 2006; Boller and Felix, 2009; Monaghan and Zipfel, 2012). Colonization of the root system of host plants with beneficial microbes is required to initiate ISR (Lugtenberg and Kamilova, 2009). Microorganisms that interact with a host plant need to avoid MTI responses if they are to effectively colonize the host (Zamioudis and Pieterse, 2012). *P. fluorescens* WCS417r can suppress flagellin-triggered MTI responses and can induce callose deposition during colonization of *Arabidopsis* (Millet et al., 2010). Callose deposition is also a central protection strategy that inhibits insects from ingesting phloem fluid (Hao et al., 2008). MAMPS and effector molecules are commonly used by ISR-inducing microbes to suppress host immunity (Zamioudis and Pieterse, 2012). For example, ISR-inducing fungus *Rhizophagus intraradices* can suppress ET- dependent defense responses utilizing the symbiotic effector SP7, thus promoting fungal biotrophy (Kloppholz et al., 2011). The 1-aminocyclopropane-1-carboxylate (ACC) deaminase produced by rhizobacteria facilitates plant development and growth, as well as mycorrhizal colonization in various crops by decreasing ET levels (Nadeem et al., 2007; Shaharoona et al., 2008; Zahir et al., 2008). Inhibition of ET perception results from the blocked activity of *BOTRYTIS-INDUCED KINASE1* (*BIK1*) localized at the plasma membrane, which acts early in defense response pathways (Veronese et al., 2006; Laluk et al., 2011). *BIK1* modulates responses of plants to phloem sap-feeding insect infestation by regulating the expression of *PAD4*, which is much higher in *bik1* mutants. The latter can induce resistance to phloem sap-feeding insects by production of ROS, cell death and leaf senescence. However, *BIK1* overexpression can render *Arabidopsis* plants more susceptible to aphid infestation (Lei et al., 2014). *PAD4* gene stimulates premature leaf senescence, which can confer resistance to aphids (Pegadaraju et al., 2005). ISR mediated by endophytic bacteria *B. velezensis* YC7010 against green peach aphid depends mainly on the elevated expression of *PAD4* with suppression of *BIK1* resulting in greater accumulation of H_2_O_2_, cell death and callose deposition in *Arabidopsis* (Rashid et al., 2017). It has been suggested that degradation of the ethylene precursor ACC by bacterial ACC deaminase, which suppresses ET-mediated immune responses like *BIK1*, results in higher expression levels of *PAD4* and *SAG13* in *Arabidopsis* colonized by bacteria. Enhanced expression of *PAD4* by the bacteria triggers more rapid H_2_O_2_ accumulation, cell death and callose deposition in plants, which can trigger ISR in the plants against insect herbivores (**Figure 2**). How beneficial rhizobacteria induce ISR against insects by suppression of plant immune responses remains unclear.

Beneficial soil microbes have coevolved with host plants for long time. Thus, they may have developed the means to overcome MTI for their mutualism to colonize host plants. Priming is a good strategy to save energy costs for even though these microbes suppress basal defense responses in the host roots (Verhagen et al., 2004; Pozo et al., 2008; Van Wees et al., 2008; Rashid et al., 2017). Primed plants display quicker and/or stronger initiation of cellular defenses when challenged by pathogen or insect attack to improve the level of defense (Conrath, 2011). ISR mediated by beneficial soil microorganisms is normally dependent on priming (Pieterse et al., 2014). Molecular changes that happen in systemic tissues upon colonization of the plant roots by beneficial microorganisms are generally minor compared to the huge molecular reprogramming triggered by pathogen or insect attack in primed plants (Pieterse et al., 2014). The primed state is frequently opaque in unchallenged plants by insects or pathogens. Priming occurs only after insect or pathogen challenge, but does not occur when the leaves are damaged by the ISR-insensitive specialist herbivore, *Pieris rapae* (Van Oosten et al., 2008). Priming mediated by the rhizobacterium *P. putida* LSW17S is based on JA, ET, and NPR1 in *Arabidopsis* plants (Ahn et al., 2007) Additionally, colonization of tomato plants by mycorrhiza can prime systemic defense responses against insect attack with increased expression of defense associated genes *allene oxide cyclase* (*AOC*), *LOXD* and *protease inhibitors* (*PI-I*, *PI-II*) (Song et al., 2013).

## Conclusion and Future Perspectives

Interactions among beneficial microbes, plants, and insects mainly involve plant growth promotion and ISR. Unlike ISR against plant pathogens, which has been well-studied for several decades, little information is available about the ISR activity against insect herbivores in related with the microbes in soil. The activation of ISR by beneficial microbes against insects through recognition of the microbes, elicitation of specific hormonal signal pathways may play vital role in plant defense responses. The biosynthesis pathways for defense related chemical compounds, enzymes, protein, secondary metabolites, and VOCs against insect herbivores can be activated by root colonization by beneficial microbes. One of the plant defense responses against insects, the accumulation of ROS scavenger peroxidases allow biosynthesis of secondary metabolites and flavonoids (especially tricin) that participate in lignin biosynthesis. The metabolic change in the biosynthesis of chemicals as direct inhibitors or repellents of insects might be also involved in physical strengthening of cell wall by lignification. This review has provided up-to-date information on the chemical changes and strengthening of physical barriers, which play important roles comprehensively in plant defense system against insect herbivores. Selecting beneficial soil microorganisms might be a more effective and cheaper way to manage the insect herbivores than development of chemical pesticides. This approach would contribute to sustainable insect pests control by development of bioproducts that would enhance plant productivity and simultaneously induce systemic resistance against insects or attractiveness to beneficial insects.

## Author Contributions

MR and YC conceived the premise for this review.

## Conflict of Interest Statement

The authors declare that the research was conducted in the absence of any commercial or financial relationships that could be construed as a potential conflict of interest.

## Abbreviations

ABA: abscisic acid
CK: cytokinin
ET: ethylene
GA: gibberellin
HIPVs: herbivore induced plant volatiles
IAA: indole-3-acetic acid
ISR: induced systemic resistance
JA: jasmonic acid
LOX2: lipoxygenase 2
MAMPs: microbe associated molecular patterns
NPR1: non-expressor of pathogenesis-related genes1
PDF1.2: plant defensin 1.2
PGPF: plant growth-promoting fungi
PGPR: plant growth-promoting rhizobacteria
POD: peroxidase
PPO: polyphenol oxidase
SA: salicylic acid
SAR: systemic acquired resistance
VOCs: volatile organic compounds.
